# Application of Adult Stem Cells in Medicine

**DOI:** 10.1155/2015/258313

**Published:** 2015-08-04

**Authors:** Pritha Ray, Abhijit De, Shahriar Yaghoubi, Aparna Khanna

**Affiliations:** ^1^Advanced Centre for Treatment, Research and Education in Cancer, Tata Memorial Centre, Kharghar, Navi Mumbai, Maharashtra 410210, India; ^2^Molecular Functional Imaging Lab, Advanced Centre for Treatment, Research and Education in Cancer, Tata Memorial Centre, Kharghar, Navi Mumbai, Maharashtra 410210, India; ^3^CellSight Technologies, San Francisco, CA 94107, USA; ^4^UCLA Department of Molecular and Medical Pharmacology, Los Angeles, CA 90095, USA; ^5^School of Science, SVKM's Narsee Monjee Institute of Management Studies, V. L. Mehta Road, Vile Parle West, Mumbai 40056, India

For any disease condition, there are two possible approaches to treatment. Treatment approaches that alleviate or suppress the disease condition are the most widespread available options for majority of diseases. This type of treatment is often applied in the form of drugs (chemical or biological) with or without surgical intervention. The second type of treatment approach reverts the pathogenic situation to normal or original state. This second option is rare, applicable to few of the diseases where drugs cannot mend the situation and are not able to restore or regenerate the normal function of the damaged tissue or organ. Regenerative remedies are commonly applied through* stem cell therapy* and to some extent by organ transplantation. Stem cells, the architect of all the structural and functional units of our body, are the utmost hope for many incurable diseases like diabetes, cardiac disease, Parkinson's, Alzheimer's, and other neurodegenerative diseases and injury related trauma. These cells depending upon their nature of origin can differentiate into many or specific kind of mature cell type to rebuild the tissue. Stem cells are present at various stages of life (embryonic, fetal, and adult). Adult stem cells have the most potential and are the safest for therapeutic utilization. Stem cell therapy is extensively used in clinic for curing hematological malignancies. However, for many other dreadful life threatening diseases, stem cell therapy is still in experimental phase and requires major effort to bring them into clinical practice.

For the last few months, we have been busy reviewing various articles sent by renowned scientists across the world for our special issue on “Application of Adult Stem Cells in Medicine.” After stringent reviews, ten articles have been selected that cover many critical aspects of stem cell therapy. They include reviews focusing on success of stem cell therapy in various diseases and original research and articles related to improvement in various aspects of stem cell therapy.

Cartilage defects and/or degeneration resulting from injury, aging, and osteoarthritis are a major cause of joint pain and disability that seriously affect quality of life. Unfortunately current surgical or pharmacological treatments can only help in temporary relief and delay in disease progression. In recent years, new strategies have been devised to repair the damaged cartilage using adult stem cells. This pressing issue of regenerating cartilage using stem cells has been elaborated by two papers in this issue. F. Perdisa et al. have discussed all recent* in vivo* studies on adipose derived mesenchymal stem cells for the treatment of articular cartilage defect. However, C. Bauge and K. Boumediene have reviewed the current status and future developments of adult stem cell therapy towards cartilage tissue engineering. Both of these papers have elucidated the promises and limitations of stem cell transplantation for cartilage defect. Readers will be aware of the major development and the specific future focus happening in this field.

Intracerebral hemorrhage (ICH) caused by sudden increase in blood pressure is not as common as ischemic stroke but is more serious and quickly causes brain and nerve damage. Depending on the location of hemorrhage and amount of injury long term treatments including physical, speech, and occupational therapy are applied but majority survive with some kind of permanent disability. In recent years, stem cell transplantation as well as cell engineering has shown promising results for various neurological diseases and regeneration of injured nerves. Application of such therapies is rare in patients suffering from ICH. J. Zhu et al., in this issue, discussed their findings on autologous bone marrow stromal cell transplantation for treating 206 patients suffering from ICH. This clinical study assessed the safety profile, feasibility, and effectiveness of surgery combined with autologous BMSC transplantation for treating ICH. Though they were unable to assess the differentiation ability of BMSC into neuronal cells and could not pursue long term followup, their study indisputably showed encouraging clinical outcome. In another review by V. Sabapathy et al., the potential of various cell therapy strategies for treating spinal cord injury has been discussed in detail with an emphasis on source of cells and appropriate preclinical models. This review highlights the insufficient data and research involved in spinal cord injury and calls for more complete and multicentric clinical trials.

Probably the most demanding area for stem cell application is the incurable neurodegenerative disorders such as Alzheimer's disease, Parkinson disease, and Huntington disease. Though etiology and symptoms of these diseases are well characterized, the underlying mechanism is yet to be understood. Therefore current treatments majorly aim towards delaying progression of the disease. Lack of appropriate animal models simulating the human pathogenesis also demands development of human disease-specific models to identify new drugs. Induced pluripotent stem cells or iPSCs have revolutionized the field of regenerative medicine and are now being utilized as models for neurodegenerative disorders to understand the biology of pathogenesis and screen novel therapeutics. Recent advancements in this newly emerging area are described in detail by W. Wan et al. in this special issue. Their manuscript presents a comprehensive review on application of iPSC in various neurodegenerative diseases narrating the promises and concerned issues.

A major thrust in stem cell research is to experiment new approaches for efficient and lineage specific differentiation of stem cells. In this special issue, three independent investigations highlight the importance of such trials for various diseases. Oliviera et al. demonstrated that priming of MSCs with endothelial growth medium improves therapeutic efficacy in the treatment of systemic arterial hypertension in a rat model. Enhanced differentiation of neuronal stem cells by Oleanolic Acid (OA) was evaluated by Y. Ning et al., who found that Nkx-2.5 transcription factor partially regulates this differentiation process. Similarly, V. Nardone et al. showed that* in vitro *Strontium treatment of hADSCs enhances cell proliferation and osteogenic differentiation through expression of early and late osteoblastic biomarkers such as ALP and HA, respectively. Their findings clearly support the use of Strontium in* in vitro* induction of bone regeneration.

The placenta, amniotic fluid, and umbilical cord are known to be rich source for neonatal MSCs. Cryopreservation of umbilical cord immediately after birth has already been commercial practice to tackle future life threatening diseases. However, such practice must be dealt with care and properly designed expreiments as discussed by O. Maslova et al. in their review.

Last but not the least, S. Kumar et al. investigated an important aspect required for final acceptance of stem cells in clinic by repetitive and noninvasive tracking of ICG labeled human placental derived MSC transplanted in live mouse.

Altogether, this special issue compiles a wide range of information on experimental and translational application of adult stem cell in regenerative medicine. We hope that contributions will generate new thoughts to readers of this journal.

